# Diverse Commensal Escherichia coli Clones and Plasmids Disseminate Antimicrobial Resistance Genes in Domestic Animals and Children in a Semirural Community in Ecuador

**DOI:** 10.1128/mSphere.00316-19

**Published:** 2019-05-22

**Authors:** Liseth Salinas, Paúl Cárdenas, Timothy J. Johnson, Karla Vasco, Jay Graham, Gabriel Trueba

**Affiliations:** a Microbiology Institute, Universidad San Francisco de Quito, Quito, Ecuador; b Department of Veterinary and Biomedical Sciences, University of Minnesota, Saint Paul, Minnesota, USA; c Mid Central Research & Outreach Center, Willmar, Minnesota, USA; d Environmental Health Sciences Division, University of California, Berkeley, California, USA; JMI Laboratories

**Keywords:** *Escherichia coli*, antibiotic resistance, clonality, plasmid analysis

## Abstract

The increased prevalence of antimicrobial resistance (AMR) among Enterobacteriaceae has had major clinical and economic impacts on human medicine. Many of the multidrug-resistant (multiresistant) Enterobacteriaceae found in humans are community acquired, and some of them are possibly linked to food animals (i.e., livestock raised for meat and dairy products). In this study, we examined whether numerically dominant commensal Escherichia coli strains from humans (*n* = 63 isolates) and domestic animals (*n* = 174 isolates) in the same community and with matching phenotypic AMR patterns were clonally related or shared the same plasmids. We identified 25 multiresistant isolates (i.e., isolates resistant to more than one antimicrobial) that shared identical phenotypic resistance patterns. We then investigated the diversity of E. coli clones, AMR genes, and plasmids carrying the AMR genes using conjugation, replicon typing, and whole-genome sequencing. All of the multiresistant E. coli isolates (from children and domestic animals) analyzed had at least 90 or more whole-genome SNP differences between one another, suggesting that none of the strains was recently transferred. While the majority of isolates shared the same antimicrobial resistance genes and replicons, DNA sequencing indicated that these genes and replicons were found on different plasmid structures. We did not find evidence of the clonal spread of AMR in this community: instead, AMR genes were carried on diverse clones and plasmids. This presents a significant challenge for understanding the movement of AMR in a community.

**IMPORTANCE** Even though Escherichia coli strains may share nearly identical phenotypic AMR profiles and AMR genes and overlap in space and time, the diversity of clones and plasmids challenges research that aims to identify sources of AMR. Horizontal gene transfer appears to play a more significant role than clonal expansion in the spread of AMR in this community.

## Abstract (precis)

Even though Escherichia coli strains may share nearly identical phenotypic AMR profiles and AMR genes and overlap in space and time, the diversity of clones and plasmids challenges research that aims to identify sources of AMR. Horizontal gene transfer appears to play a more significant role than clonal expansion in the spread of AMR in this community.

## INTRODUCTION

Antimicrobial resistance (AMR), especially among Enterobacteriaceae, constitutes an increasing threat to global health (1, 2). Some of the bacterial AMR found in humans has been linked to food-animals (i.e., livestock raised for meat and dairy products) (3). Studies have documented that antimicrobial use in food animal production is a regular practice in Ecuador and many other countries across the globe (4–7) and that this use increases the likelihood of both the presence of multidrug-resistant (multiresistant) bacteria in the human microbiota and horizontal gene transfer of AMR genes to human microbiota (8–10).

Increases in AMR may be greater in low- and middle-income countries (LMICs) than in high-income countries, in part because of regulations controlling the use of antimicrobials for humans and food animals are lacking or not enforced (11, 12); in countries like Ecuador, antimicrobials can be purchased over the counter without prescriptions. Additionally, contact with food animal waste, a potential reservoir of drug-resistant bacteria and mobile genetic elements associated with AMR genes (13), can be higher in food-animal-producing regions of LMICs than industrialized countries as untreated food animal wastes are often used to fertilize crops (14). Most research on AMR transmission associated with food animals has focused on commercial-scale production (3, 15, 16), and little research has focused on small-scale food animal production, which is increasingly found to use antimicrobials (15). Despite the potential of small-scale food animals to transmit AMR in a community (17–20), this connection is poorly understood.

Understanding the potential for small-scale food animal production to spread AMR to human microbiota is critical (21, 22), since it represents an important yet underappreciated reservoir of AMR genes. In this sense, the study of small-scale food animal production in LMICs could be a model for studying AMR dissemination between different sources (23). A study looking at AMR genes in a community suggests that a person’s habitat explains the variation in AMR carriage and that AMR is significantly correlated with the composition of the community and not “randomly distributed across habitats” (19).

Escherichia coli is an important species associated with the AMR crisis because it can evolve from a commensal, drug-susceptible state to become multidrug resistant and can cause opportunistic infections (24). Due to its abundance in the intestine, its ability to grow in fecal matter outside the host, and its ability to colonize different hosts, E. coli is probably among the most common members of the microbiota transmitted between warm-blooded animals (25). E. coli is also very active in the horizontal transfer of AMR genes to other bacteria (26). The majority of previous studies examining AMR E. coli have analyzed colonies isolated in antimicrobial-containing media. In this study, we analyzed numerically dominant E. coli strains, defined as the E. coli clones that were present in the highest proportion, obtained from fecal samples from children and domestic animals and isolated in plates without antibiotics (27). The goal of this study was to better understand the E. coli population dynamics in a community and how AMR can spread within the community. Understanding how E. coli clones and AMR genes spread in a community between humans and domestic animals has the potential to inform policies that aim to mitigate the rise in AMR.

## RESULTS

Two hundred thirty-seven E. coli isolates were recovered: 63 from child fecal samples and 174 from domestic animals. More than one-third of the isolates (38.4%) were susceptible to all 12 antimicrobials evaluated: 46 (19.4%) were resistant to one antimicrobial, 19 (8.0%) to two antimicrobials, and 81 (34.2%) to three or more antimicrobials.

### Antimicrobial resistance in humans and domestic animals.

In general, isolates obtained from children showed higher phenotypic resistance than isolates from domestic animals. Human isolates had statistically significant higher resistance to amoxicillin-clavulanate, ampicillin, streptomycin, sulfisoxazole, and trimethoprim than animal isolates (Table 1). The highest percentages of phenotypic resistance in isolates obtained from children were to tetracycline (50.8%), sulfisoxazole (49.2%) and ampicillin (49.2%), and the highest in isolates from domestic animals were to tetracycline (39.7%), sulfisoxazole (24.1%), and cephalothin (23%), a first-generation cephalosporin (Table 1). The most frequent multiresistance profile, found in 13.6% of the isolates, was tetracycline–sulfisoxazole–ampicillin–streptomycin–trimethoprim-sulfamethoxazole; the majority of these isolates belonged to humans (63.6%) (Table 2; see Table S1 in the supplemental material).

**TABLE 1:** Prevalence of phenotypically resistant E. coli found in fecal samples from children and domestic animals

Antimicrobial(s)	No. (%) of samples from:		Chi-square test^*a*^	*P* value^*a*^
	Children (*n* = 63)	Domestic animals (*n* = 174)		
Amoxicillin-clavulanate	6 (9.5)	4 (2.3)	**5.970**	**0.015**
Ampicillin	31 (49.2)	35 (20.1)	**19.480**	**<0.001**
Cefotaxime	4 (6.4)	10 (5.8)	0.030	0.862
Cephalothin	20 (31.8)	40 (23.0)	1.876	0.171
Chloramphenicol	6 (9.5)	32 (18.4)	2.701	0.100
Ciprofloxacin	4 (6.4)	16 (9.2)	0.485	0.486
Gentamicin	2 (3.2)	2 (1.2)	1.143	0.285
Imipenem	0 (0)	2 (1.2)		
Streptomycin	26 (41.3)	31 (17.8)	**13.929**	**<0.001**
Sulfisoxazole	31 (49.2)	42 (24.1)	**13.637**	**<0.001**
Tetracycline	32 (50.8)	69 (39.7)	2.347	0.126
Trimethoprim-sulfamethoxazole	27 (42.9)	36 (20.7)	**11.646**	**<0.001**

a Values in boldface are statistically significant (*P* < 0.05).

**TABLE 2:** Genetic characteristics and phenotypic antimicrobial resistance patterns of multiresistant E. coli isolates and transconjugants^*a*^

Isolate ID	Origin	MLST result with^*b*^:		Isolate AMR profile^*c*^	AMR genes	Plasmid(s)	pMLST	Transconjugant AMR profile^*d*^	Replicon(s)^*g*^
		7 genes	8 genes						
47	Child	ST517		TE-G-SXT-S-AM-CF	*bla*_TEM-1B_, *dfrA8*, *qnrB19*, *strA*, *strB*, *tetB*	IncFII(pRSB107), IncFIB(AP001918)	FII43, FIB11	TE-G-SXT-S-AM-CF	L, P, X3, FIIS, FIC, FII
52	Child	ST349		TE-G-SXT-S-AM-CF	*bla*_TEM-1B_, *dfrA7*, *dfrA8*, *strA*, *strB*, *sul1*, *sul2*, *tetA*	IncFII(pHN7A8), IncFII, IncQ1	FI33, FIB29	TE-G-SXT-S-AM-CF	P, I1γ, FIIS, FIC, FII
145	Child	ST4577		TE-G-SXT-S-AM-CF	*bla*_TEM-1B_, *dfrA8*, *strA*, *strB*, *sul2*, *tetB*	IncFIB(pLF82), IncFII(pHN7A8)	FII11	TE-G-SXT-S-AM-CF	FIA, W, A/C, FIIS, X2, FII
157	Child	ST226	ST681	TE-G-SXT-S-AM-CF	*aadA1*, *bla*_TEM-1B_, *dfrA15*, *qnrB19*, *tetA*	IncFII(pSE11), IncFIB(AP001918), IncFII, Col(MG828)	FI79, FIB28	TE-G-SXT-S-AM-CF	P, A/C, FIIS, X2, FII
159	Child	ST226	ST681	TE-G-SXT-S-AM-CF-C-CTX-AMC*	*aadA1*, *bla*_TEM-1B_, *dfrA15*, *qnrB19*, *sul1*, *tetA*	IncFII(pSE11), IncFIB(AP001918), IncFII, Col(MG828)	FI79, FIB28	TE-G-SXT-S-AM-CF-C-CTX-AMC^*e*^	P, A/C, FIIS, X2, FII
								TE-S-AM-AMC^*e*^	A/C, FIIS, X2, FII
211	Chicken	ST8061	ST305	TE-G-SXT-S-AM-CF-C-CTX-AMC-CIP*	*aadA1*, *bla*_CMY-2_, *bla*_TEM-1B_, *catA1*, *cmlA1*, *floR*, *fosA*, *lnuF*, *qnrB19*, *strA*, *strB*, *sul2*, *sul3*, *tetA*	IncB/O/K/Z, IncFII, IncFIB(AP001918), IncQ1	FII64, FIB27		FIB, L, P, FIIS, FII^*h*^
								TE-G-SXT-S^*f*^	FIB, FIIS, FII
191	Pig	ST8061	ST305	TE-G-SXT-S-AM-CF-C-CTX-AMC-CIP*	*bla*_CMY-2_, *bla*_TEM-1B_, *catA1*, *qnrB19*, *strA*, *strB*, *sul2*, *tetA*	IncFIB(AP001918), IncFII, IncB/O/K/Z, IncQ1	FII64, FIB27	TE-G-SXT-S-AM-CF-C-CTX-AMC-CIP	FIB, L, P, A/C, FIIS, FII
58	Chicken	ST189	STNEW4	TE-G-SXT-AM-C-CIP†	*dfrA14*, *strA*, *strB*, *sul2*	IncI2, IncY	FI43	Not conjugated	I2, L, A/C, FIIS, FII^*i*^
132	Chicken	ST48		TE-G-SXT-AM-C†	*aadA1*, *aadA2*, *bla*_TEM-1B_, *cmlA1*, *dfrA12*, *dfrA14*, *mef*, *qnrB19*, *strA*, *strB*, *sul2*, *sul3*, *tetA*, *tetB*	IncFII(29), IncFIB(K), IncFIA(HI1)	FII29, FIA13	TE-G-SXT-AM-C	L
19	Dog	ST101		TE-G-SXT-S-AM	*aadA2*, *bla*_TEM-1B_, *dfrA12*, *sul3*, *tetA*, *tetM*	IncFII, IncN3	FII34	TE-G-SXT-S-AM	L, FIIS, Y, FII
44	Cat	ST10	ST2	TE-G-SXT-S-AM	*bla*_TEM-1B_, *dfrA8*, *strA*, *strB*, *sul2*, *tetB*	IncFIB(AP001918), IncFII(pRSB107)	FII1, FIB54	TE-G-SXT-S-AM	L, FIIS, Y, FII
90	Pig	STNEW1		TE-G-SXT-S-AM	*bla*_TEM-1B_, *dfrA8*, *strA*, *strB*, *sul2*, *tetB*	IncFII(pRSB107), IncFIB(AP001918), IncI1	FII48, FIB25	TE-G-SXT-S-AM^*e*^	L, FIIS, Y, FII
								TE-G-S-AM^*e*^	I2, FIB, L, P, FIIS, Y, FII
113	Child	ST226	ST681	TE-G-SXT-S-AM	*bla*_TEM-1B_, *dfrA8*, *strA*, *strB*, *sul2*, *tetA*	IncFIB(AP001918), IncFII(pRSB107), IncFII(29)	FII1, FIB54	TE-G-SXT-S-AM^*e*^	I2, FIB, P, FIIS, Y, FII
								TE-G-S-AM^*e*^	I2, FIIS, FII
169	Child	STNEW2		TE-G-SXT-S-AM	*bla*_TEM-1B_, *dfrA8*, *strA*, *strB*, *sul2*, *tetB*	IncFIB(AP001918), IncFII, IncFII(pRSB107)	FII1, FIB54	TE-G-SXT-S-AM	FIIS, FII
200	Child	ST10	ST767	TE-G-SXT-S-AM	*aadA5*, *bla*_TEM-1B_, *dfrA17*, *mphA*, *strA*, *strB*, *sul1*, *sul2*, *tetA*	IncFIB(AP001918), IncFIA, IncFII(pCoo)	FII10, FIA2, FIB20	TE-G-SXT-S-AM	FIB, FIA, A/C, FIIS, FII
202	Child	ST2952		TE-G-SXT-S-AM	*bla*_TEM-1B_, *dfrA8*, *strA*, *strB*, *sul2*, *tetA*	IncQ1, IncFII(pCoo)	FII16	TE-G-AM-SXT-S	FIIS, FII
203	Child	ST10	ST2	TE-G-SXT-S-AM	*bla*_TEM-1B_, *dfrA5*, *strA*, *strB*, *sul1*, *sul2*, *tetB*	IncFII(pRSB107), IncB/O/K/Z, IncI2	FII6	TE-G-SXT-S-AM^*e*^	FIIS, FII
								TE-G-S-AM^*e*^	I2, L, FIIS, FIC, FII
212	Chicken	ST394		TE-G-SXT-S-AM	*aadA1*, *bla*_TEM-1B_, *dfrA1*, *strA*, *strB*, *sul1*, *sul2*, *tetA*	IncFII(pHN7A8), IncFII, Col(BS512)	FII11	TE-G-SXT-S-AM	I1α, FIB, FIA, P, FIIS, FII
233	Child	ST131		TE-G-SXT-S-AM	*bla*_TEM-1B_, *dfrA8*, *strA*, *strB*, *sul2*, *tetB*	IncFIB(AP001918), IncFII, IncFIC(FII), IncFIA, IncFII(pRSB107), IncI1, Col(BS512)	FII1, FIA1, FIB1	TE-G-SXT-S-AM^*e*^	I2, FIIS, FIC, FII
								TE-G-S-AM^*e*^	FIIS, FIC, FII
71	Child	STNEW3		TE-G-SXT	*aadA5*, *dfrA17*, *qnrB19*, *sul2*, *tetA*	p0111	NEW1	TE-G-SXT	I1γ, FIIS, FII
253	Child	ST3075		TE-G-SXT	*dfrA14*, *strA*, *strB*, *sul2*, *tetA*	IncFII(pCoo), IncY, IncB/O/K/Z	FII43, FIB24	TE-G-SXT	B/O, I1γ, A/C, FIIS, FIC, FII
50	Guinea pig	ST189	STNEW5	TE-G-SXT-S-CIP‡	*aadA24*, *dfrA14*, *dfrA15*, *strA*, *strB*, *sul1*, *sul2*	IncFIB(AP001918), IncI1, IncI2, Col156	FII17	TE-G-SXT-S	I1α, FIB, P, FII
226	Chicken	ST155		TE-G-SXT-S‡	*dfrA14*, *qnrB19*, *strA*, *strB*, *sul2*, *tetA*	IncFII(29)	FII29	TE-G-SXT-S	FIIS, FII
241	Child	ST10	ST2	TE-G-SXT-S‡	*dfrA5*, *qnrB19*, *strA*, *strB*, *sul1*, *sul2*, *tetA*	IncB/O/K/Z, Col(MG828)	NEW2	TE-G-SXT-S^*e*^	P, K
								TE-G-S^*e*^	P, K
102	Child	ST1196		TE-G-SXT-S-AM-CF-C-CIP	*aadA1*, *aadA2*, *bla*_TEM-1B_, *cmlA1*, *dfrA12*, *floR*, *fosA*, *lnuF*, *sul3*, *tetA*	IncFIB(AP001918), IncFII(29), p0111	FII29, FIB1	TE-G-SXT-S-AM-CF-C-CIP	FIB, FIIS, FII

a AMR genes, plasmids, and pMLST were obtained from WGS.

b MLST profiles were obtained from https://cge.cbs.dtu.dk/services/MLST/.

c Profiles linked by the same symbol (*, †, or ‡) indicate that three antimicrobial resistance patterns, including isolates differing only in CIP, were considered a single multiresistance pattern because this resistance can generally be associated with chromosomal elements. However, in some isolates, the resistance was transferred into transconjugants. The antimicrobial compounds are abbreviated as follows: AMC, amoxicillin-clavulanate; AM, ampicillin; CTX, cefotaxime; CF, cephalothin; C, chloramphenicol; CIP, ciprofloxacin; S, streptomycin; G, sulfisoxazole; TE, tetracycline; SXT, trimethoprim-sulfamethoxazole.

d Most isolates transferred resistance patterns.

e Some isolates transferred complete and partial patterns.

f One isolate transferred only a partial pattern.

g Replicons were characterized from transconjugants.

h Result of replicon typing referred to donor strain, because we obtained only a partial transconjugant.

i Result of replicon typing referred to donor strain, because there were no transconjugants obtained.

10.1128/mSphere.00316-19.2 TABLE S1 Halo diameters (mm) of isolates and their transconjugants. Download Table S1, DOCX file, 0.02 MB. Copyright © 2019 Salinas et al. 2019 Salinas et al. This content is distributed under the terms of the Creative Commons Attribution 4.0 International license.

### Bacterial conjugation.

For the bacterial conjugation assays, we selected 15 multiresistant isolates from children and 10 multiresistant isolates from domestic animals. All of the isolates shared the same multiresistance phenotypic pattern (tetracycline–sulfisoxazole–trimethoprim-sulfamethoxazole), as described above; this pattern always was transferred in the conjugation assays. We obtained a total of 30 transconjugants from 24 isolates. Twenty-three isolates transferred their complete phenotypic resistance pattern to the receptor bacteria; six of these also transferred a partial phenotypic pattern—i.e., resistance to some of the antimicrobials of the complete phenotypic resistance pattern. From one isolate, we obtained only a transconjugant with partial donor phenotypic pattern (Table 2 and Table S1).

### Isolate genotyping.

Among the 25 E. coli isolates selected for the bacterial conjugation, we identified 18 different sequence types (STs): seven STs were found in domestic animals only, 10 STs were found in isolates from children only, and one was present in isolates from both sources (Table 2).

Among animals, an isolate from a guinea pig and another from a chicken belonged to ST189; however, extended multilocus sequence typing (MLST) assigned them to novel STs STNEW4 and STNEW5, respectively. Two isolates (1 from a chicken and 1 from a pig) belonged to ST8061, and extended MLST assigned them both to ST305; whole-genome sequencing (WGS), however, showed that the isolates were not identical (275 single nucleotide polymorphism [SNP] differences). Among the human isolates, 3 belonged to ST226, and extended MLST found them to belong to ST681. Again, WGS showed two isolates were not identical (90 SNP differences), but they were more closely related than the other isolates (3,601 and 3,608 SNP differences, respectively). Three human isolates and 1 cat isolate belonged to ST10; extended MLST analysis showed that these 2 isolates from humans and the isolate from the cat belonged to ST2, while the remaining human isolate was ST767 (Table 2). Whole-genome sequencing, however, showed that two human isolates (ST2) were not related (5,932 SNP differences), and these two human isolates differed from the cat isolate by 6,138 and 7,409 SNPs, respectively.

### Plasmid genotyping.

We identified 17 replicon types in the transconjugants: 7 replicon types (X3, FIC, I1γ, W, X2, B/O, and K) originated in human isolates, 9 (L, P, FIIS, FII, FIA, A/C, γ, I2, and FIB) were present in isolates from both human and domestic animals, and one (I1α) was present in only one isolate from an animal. The most common replicons were FII and FIIS, which were found in 23 of the isolates (92%) and 22 (88%), respectively (Table 2).

**FIG 1:**
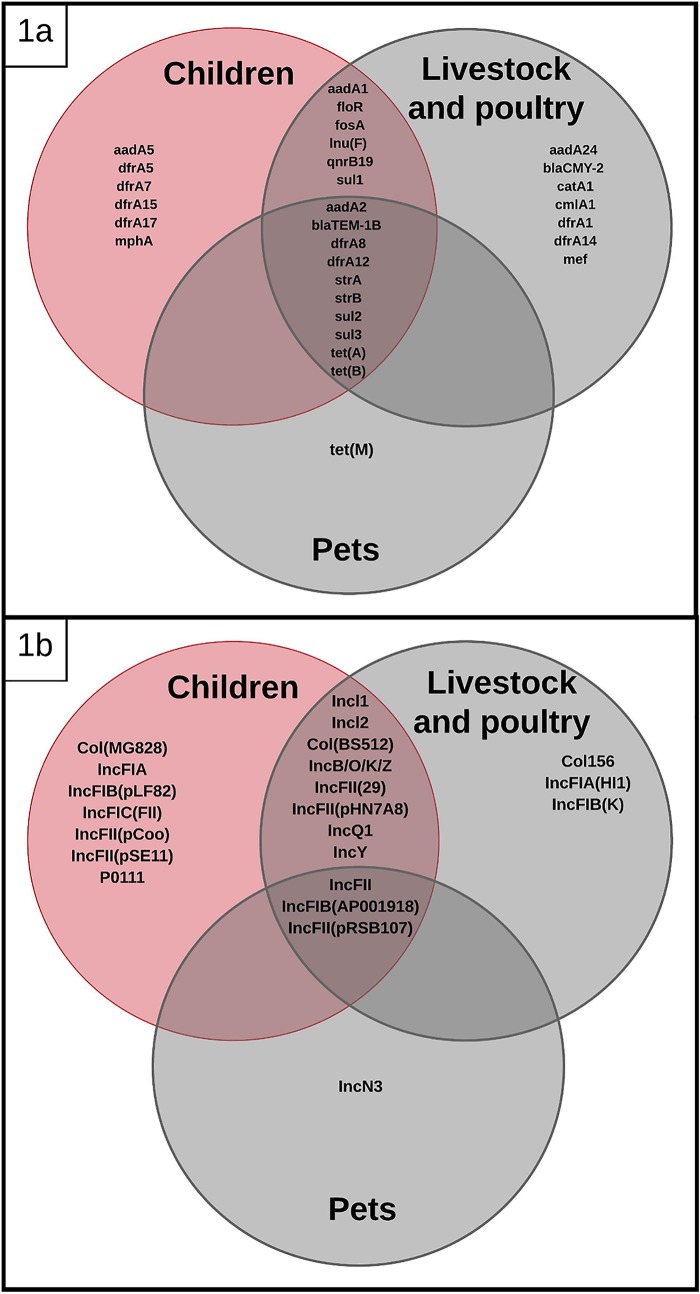
Venn diagrams showing shared antimicrobial resistance genes (a) and replicons (b) among E. coli isolates from children, livestock, poultry, and pets.

Whole-genome sequencing of the 25 selected isolates identified 22 replicons, and 11 replicons were found in isolates from both children and domestic animals: IncFII(pRSB107), Incl1, IncQ1, IncFII(29), IncY, IncFII, IncB/O/K/Z, Incl2, IncFIB(AP001918), IncFII(pHN7A8), and Col(BS512). Four replicons were found only in animal isolates [IncN3, IncFIA(HI1), IncFIB(K), and Col156], and 7 replicons were found only in human isolates [IncFII(pSE11), IncFII(pCoo), IncFIC(FII), IncFIB(pLF82), Col(MG828), IncFIA, and p0111] (Fig. 1a and Table 2).

Twenty-eight F-type plasmids were further characterized by pMLST: 8 of them (FII64, FIB27, FI43, FIA13, FII34, FII48, FIB25, and FIB24) came from animal samples, and 15 (FIB11, FI33, FI79, FIB28, FII10, FIA2, FIB20, FII16, FII6, FIA1, FII17, FIB1, FIB29, NEW1, and NEW2) were from human samples, while 5 (FII43, FII11, FII29, FII1, and FIB54) were identified in both sources. The replicon type did not appear to correlate with a specific phenotypic resistance pattern (Table 2). Further characterization of plasmids using long-read PacBio sequencing demonstrated that none of the plasmids compared were identical based on pMLST or whole-plasmid sequence alignments; however, there were similarities in AMR genes. For example, the association of Tn*2*(*bla*_TEM-1B_) (with an identical DNA sequence) was encountered in 4 different plasmids in 4 different strains from children in different households. The DNA sequences of Tn*2*(*bla*_TEM-1B_) were identical to others found in other bacterial species in GenBank, suggesting that this association is not novel. Additionally, two different STs from two children in different households had plasmids sharing 73% of gene content but different replicon types (see Fig. S1 in the supplemental material).

10.1128/mSphere.00316-19.1 FIG S1 Plasmid 6 (from transconjugant 145) and plasmid 4 (from complete transconjugant 233) alignments using Mauve and gene annotations on Prokka and ISFinder showing shared genomic structures between two different plasmids (different replicon types). Shared genes are colored in green and red, replicon names and locations are in blue boxes, and transposons/insertion sequences are in clear boxes. Download FIG S1, TIF file, 1.0 MB. Copyright © 2019 Salinas et al. 2019 Salinas et al. This content is distributed under the terms of the Creative Commons Attribution 4.0 International license.

### Analysis of antimicrobial resistance genes.

Whole-genome sequencing of the 25 selected isolates showed 30 allelic variants of antimicrobial resistance genes. The following AMR genes were found in children and domestic animals: *bla*_TEM-1B_, *dfrA8*, *dfrA12*, *dfrA14*, *dfrA15*, *qnrB19*, *strA*, *strB*, *tetA*, *tetB*, *sul1*, *sul2*, *sul3*, *floR*, *aadA1*, *aadA2*, *cmlA1*, *inuF*, and *fosA* (Fig. 1b). AMR genes found only in domestic animal isolates included *bla*_CMY-2_, *catA1*, *mef*, *tetM*, *dfrA1*, and *aadA24*, and AMR genes found only in children included *dfrA5*, *dfrA7*, *dfrA17*, *aadA5*, and *mphA* (Table 2). Phylogenetic analysis of the most common genes showed that *tetA*, *tetB*, and *dfrA8* were identical, and we found sequences classified as *aadA1*-like, *strA*-like, *strB*-like, and *sul2*-like with SNPs clustering independently from strain origin (data not shown).

## DISCUSSION

In this semirural community, we found that numerically dominant commensal E. coli strains (showing similar antimicrobial resistance and same antibiotic resistance genes) colonizing children and domestic animals in the same period of time and in the same community are genotypically diverse. We also found that plasmids carrying the same antibiotic resistance genes were distinct, which is consistent with recent reports showing that AMR genes move frequently among different plasmids (28, 29). Our research suggests that a common pool of AMR genes could be cocirculating on different plasmids among different E. coli clones in a community (Table 2)—probably through dissemination mediated by transposons, integrons, or gene cassettes (28, 30). Even when the same resistance gene alleles and same plasmid replicon types were identified across isolates, the plasmids harboring these traits were still distinct. We also found potential evidence of Tn*2* participation in mobility of the gene *bla*_TEM-1B_, as we found this transposon-gene association in 4 different plasmids and 4 genetically different E. coli strains. This is indicative of common pools of transposable elements actively moving genes among different plasmids (31). The findings of this study should raise caution about the conclusions reached in many studies, which have used MLST and replicon typing to identify the sources of AMR genes and have concluded that matching MLST profile and AMR gene profile suggests clonality (32–34).

Previous studies have found that some numerically dominant E. coli strains from domestic animals and humans can be shared within the same household (35–38), although we did not find direct evidence of clone sharing within households enrolled in this study. The reason for this discrepancy may be that we selected individuals in a community instead of individuals in the same household.

Other reports have shown that whole-genome sequences of isolates from food animals and human bloodstream infections with similar AMR patterns (both groups of isolates collected in different times and probably from different communities) were different and did not share plasmids. The authors concluded that there is no evidence of AMR transmission from food animals to humans (39). In our study, we collected samples during the same period of time and from a small community; however, we were not able to find recent clonal relatedness even among human isolates. Our results suggest that there is substantial diversity of E. coli clones (and plasmids) even in small communities; the possibility of finding the same clones isolated in different time frames and from different communities seems very low. Other studies using strains isolated during the same period have succeeded in finding close clonal relatedness between humans and chickens (59).

We showed evidence that human and domestic animal strains share the same replicons and pMLST profiles: IncFII, IncFII(29), IncFII(pRSB107), IncFII(pHN7A8), IncFIB(AP001918), Incl1, Incl2, IncQ1, IncY, IncB/O/K/Z, Col(BS512), FII1, FII11, FII29, FII43, and FIB54. This would suggest that plasmids were shared among numerically dominant and antimicrobial-resistant E. coli strains from humans and domestic animals in this community. However, the long-read sequencing of plasmids indicated that these plasmids were not identical. Still, allelic variants of some antimicrobial resistance genes were identical among isolates from humans and domestic animals. This again suggests that mobile genetic elements within these diverse plasmids, such as transposons, conjugative transposons, and integrons, may be more actively involved in the mobility of AMR genes between plasmids and bacterial cells than plasmid transfer itself (31, 40, 41). Also our data suggest that many of the plasmids circulating in E. coli (in the same human community) could share many genes (including AMR genes and replicons), but they are not the same.

This remarkable genetic plasticity has been described in some plasmids carrying multiple resistance genes (31, 42). Our report suggests that assessment of the role of domestic animals in the current AMR crisis is a very complex endeavor that will be accomplished only through the use of powerful DNA sequencing technology (34) and the understanding of E. coli evolution and the population structure of E. coli in domestic animals and humans. Nevertheless, it is too early to discard the link between antimicrobial use in domestic animals and the current AMR crisis. The results described here, although they are from a study of a small community, could serve as a model of microbiological transmission in the animal-human interface. We also provide evidence that caution should be exercised when assessing the AMR gene linkage of E. coli populations from different animal species.

## MATERIALS AND METHODS

### Study location.

The study was carried out in the semirural community of Otón de Vélez, in the parish of Yaruquí, located at an altitude of approximately 2,500 m above sea level, east of the capital city of Quito. Inhabitants in this community commonly practice small-scale food animal production. Sixty-five households were recruited randomly and were enrolled in the study if they met the inclusion criteria. Inclusion criteria for the households were (i) households with a primary child care provider present who was over 18 years of age, (ii) a child present in the household between the ages of 6 months and 5 years, and (iii) informed consent provided by a primary child care provider to participate in the study. Stool samples were obtained once from 64 children, and 203 fecal samples were obtained from 12 species of domestic animals. One individual representative of each animal species found in each household was tested. Sixty-eight percent of households had chickens, 64.5% had guinea pigs (raised for food), 64.5% had dogs, 58% had pigs, 32.3% had rabbits, 11.3% had cattle, 11.3% had cats, 10% had ducks, 8% had quail, 5% had sheep, 3% had geese, and 1.6% had horses.

### Ethical considerations.

The study protocol was approved by the Institutional Animal Care and Use Committee at the George Washington University (IACUC no. A296), as well as the Bioethics Committee at the Universidad San Francisco de Quito (no. 2014-135M) and the George Washington University Committee on Human Research Institutional Review Board (IRB no. 101355).

### Sample collection.

A single fecal sample was collected from children less than 5 years of age and from livestock, poultry, and pets living in the children’s household from June to August 2014. Stool samples from the children were collected by the child’s primary caretaker. Infant caretakers were instructed to cover the inside of the diaper with a clean plastic lining; for older children; fecal collection containers were provided as well as a plastic liner to place under the toilet seat to catch the stool and avoid collecting the sample from the toilet bowl. Participants were instructed to keep the sample container double-bagged in the refrigerator until field staff could pick the sample up the same morning (43). Animal fecal matter was collected by the study team from the environment where the animals defecated, avoiding potential contamination with other residues. The samples were transported in a cooler (approximately 4°C) to the laboratory and were processed within 8 h of collection.

### E. coli isolation.

Fecal samples were streaked onto MacConkey agar (Difco, Sparks, Maryland) and incubated at 37°C for 18 h without antimicrobials, after which five lactose-positive colonies were selected (27). The colonies were transferred to Chromocult coliform agar (Merck KGaA, Darmstadt, Germany) for the putative identification of Escherichia coli through its β-d-glucuronidase activity and confirmed with API RapiD-20E (bioMérieux, Marcy l’Etoile, France) identification percentages of greater than 95%. One E. coli isolate from each fecal sample was preserved at −80°C in brain heart infusion medium (Difco, Sparks, MD) with 20% glycerol.

### Antimicrobial susceptibility testing.

Each isolate was regrown on nutrient agar (Difco, Sparks, MD) at 37°C for 18 h for evaluation of antimicrobial susceptibility by the disk diffusion method using Mueller-Hinton agar (Difco, Sparks, MD) according to the resistance or susceptibility interpretation criteria from Clinical and Laboratory Standards Institute (CLSI) guidelines (44). E. coli ATTC 25922 was used as the quality control strain. Antimicrobials used for susceptibility testing included the following (with the corresponding abbreviations used in Table 2 in parentheses): amoxicillin-clavulanate (AMC; 20/10 μg), ampicillin (AM; 10 μg), cefotaxime (CTX; 30 μg), cephalothin (CF; 30 μg), chloramphenicol (C; 30 μg), ciprofloxacin (CIP; 5 μg), gentamicin (10 μg), imipenem (10 μg), streptomycin (S; 10 μg), sulfisoxazole (G; 250 μg), tetracycline (TE; 30 μg), and trimethoprim-sulfamethoxazole (SXT; 1.25/23.75 μg) (44).

### Bacterial conjugation.

Following antimicrobial susceptibility testing, we selected 25 multidrug-resistant isolates that shared antibiotic resistance to 3 antimicrobials, TE-G-SXT (which was the most common pattern of multiresistance), although some isolates showed additional antimicrobial resistance (Table 2); this was the most common multiresistance pattern in the community. Isolates were selected based on similar phenotypic multiresistance patterns in isolates from domestic animals and humans. Each of the 25 multiresistant isolates was used as a donor strain for the conjugation assay, with three different strains as receptors: E. coli J53 resistant to sodium azide, E. coli TOP10 (Invitrogen, Carlsbad, CA, USA) resistant to rifampin, and E. coli TOP10 resistant to nalidixic acid. Selection of mutant E. coli TOP10 resistant to rifampin and nalidixic acid was carried out as previously described (45). Prior to each experiment for conjugation, the donor and recipient strains were inoculated in 10 ml of Trypticase soy broth (Difco, Sparks, MD) and grown at 37°C for 18 h, and the strains in the logarithmic growth phase were mixed and incubated at 37°C for 18 h. For the selection of transconjugants, 100 μl of the mixture was inoculated by the spread plate method onto nutrient agar supplemented with tetracycline (15 μg/ml) and one of the following antimicrobials according to the recipient strain used (45): sodium azide (200 μg/ml) (46), rifampin (100 μg/ml), or nalidixic acid (30 μg/ml). Tetracycline was used as the donor selector, since this resistance was present in all 25 multiresistant isolates. The transconjugants were evaluated for the 12 antimicrobials tested by the disk diffusion method in order to determine the acquired resistance. The transconjugants were stored as described above.

### DNA extraction.

Total DNA was extracted from the same 25 isolates showing similar AMR phenotypic profiles using the DNeasy Blood & Tissue kit (Qiagen, Hilden, Germany) following the manufacturer's recommendations. Plasmid DNA from the transconjugants was extracted using the QIAprep Spin Miniprep kit (Qiagen, Hilden, Germany) following the manufacturer's recommendations.

### Replicon typing.

Replicon typing of transconjugants was carried out from plasmid DNA using a PCR-based replicon typing kit (PBRT kit; Diatheva, Cartoceto, Italy) following the manufacturer’s instructions (47).

### DNA sequencing.

For the 25 isolates selected, whole-genome sequencing (WGS) was performed using Illumina MiSeq. Sequencing was performed at the University of Minnesota Mid-Central Research and Outreach Center (Willmar, MN) using a single 250-bp dual-index run on an Illumina MiSeq with Nextera XT libraries to generate approximately 30- to 50-fold coverage per genome. Several transconjugants were also sequenced using PacBio technology at the University of Minnesota Genomics Center (Minneapolis, MN). SMRTbell template libraries were generated from previously isolated unsheared raw genomic DNA using the Pacific Biosciences SMRTbell template preparation kit 1.0 (Pacific Biosciences, Menlo Park, CA). Finished DNA libraries subsequently were subjected to DNA size selection using the BluePippin DNA size selection system (Sage Science, Inc.) with a 7-kb cutoff to select DNA fragments larger than 7 kb. Sequencing was performed on the PacBio Sequel (Pacific Biosciences, Menlo Park, CA).

### Data analysis.

Illumina raw reads were quality-trimmed and adapter-trimmed using trimmomatic (48). Reads were assembled using the SPaDES assembler (49). PacBio reads were assembled using canu (50). The contigs obtained were then annotated with Prokka (51). Resistance genes (ResFinder 2.1), Gram-negative plasmid types (PlasmidFinder 1.3), plasmid allele types (pMLST; pMLST 1.4), and multilocus sequence typing (MLST) profiles, based on the sequences of internal fragments of the 7 housekeeping genes *adk*, *fumC*, *gyrB*, *icd*, *mdh*, *purA*, and *recA* and extended MLST with the additional 8 housekeeping genes *dinB*, *icdA*, *pabB*, *polB*, *putP*, *trpA*, *trpB*, and *uidA* (for isolates that shared a profile based on MLST of 7 genes) (MLST 1.8) were obtained from the WGS using the Center for Genomic Epidemiology tools (http://www.genomicepidemiology.org/) (52). Phylogenetic analysis of individual genes/segments was performed using MEGA7 (53), and WGS alignments were performed using Mauve (54, 55). Insertion sequence elements were identified using ISFinder (56). Plasmid maps were constructed using XPlasMap version 0.96 (http://www.iayork.com/XPlasMap/). Genetic relatedness between isolates, taking in account the mutation rate of E. coli under natural conditions (∼1 to 20 SNPs per year) (28, 57), was assessed by SNP analysis using Snippy (58). Significant differences (*P* < 0.05) between phenotypic AMR prevalence of isolates obtained from children and isolates obtained from domestic animals were tested using a chi-square test.
