# Supplementary material for: Diverse Commensal Escherichia coli Clones and Plasmids Disseminate Antimicrobial Resistance Genes in Domestic Animals and Children in a Semirural Community in Ecuador
Source: mSphere. 2019 May 22;4(3):e00316-19. doi: 10.1128/mSphere.00316-19 (PMC6531886; doi:10.1128/mSphere.00316-19)
Supplement: TABLE S1 [file mSphere.00316-19-st001.docx]

**SUPPLEMENTAL MATERIALS Table S1.** Halo diameters of isolates and their transconjugants (mm)

| **Isolate ID** | **AMC** | **AM** | **CTX** | **CF** | **C** | **CIP** | **CN** | **IPM** | **S** | **G** | **TE** | **SXT** |
| --- | --- | --- | --- | --- | --- | --- | --- | --- | --- | --- | --- | --- |
| 47 | 14 | **0** | 28 | **13** | 24 | 31 | 19 | 25 | **0** | **0** | **0** | **0** |
| Complete transconjugant | 14 | 0 | 35 | **11** | 30 | 33 | 22 | 33 | **0** | **0** | **0** | **0** |
| 52 | 14 | **0** | 28 | **11** | 23 | 24 | 22 | 28 | **0** | **0** | **0** | **0** |
| Complete transconjugant | 14 | 0 | 40 | **12** | 23 | 35 | 25 | 33 | **0** | **0** | **0** | **0** |
| 145 | 17 | **0** | 32 | **14** | 21 | 35 | 19 | 31 | **0** | **0** | **0** | **0** |
| Complete transconjugant | 17 | **0** | 40 | **10** | 30 | 35 | 25 | 33 | **0** | **0** | **0** | **0** |
| 157 | 17 | **0** | 33 | **11** | 26 | 26 | 20 | 27 | **9** | **0** | **0** | **0** |
| Complete transconjugant | 19 | **0** | 38 | **12** | 31 | 32 | 23 | 34 | **0** | **0** | **0** | **0** |
| 159 *^a^* | **0** | **0** | **18** | **0** | **0** | 21 | 20 | 27 | **10** | **0** | **0** | **0** |
| Complete transconjugant | **0** | **0** | **21** | **0** | **11** | 31 | 29 | 32 | **0** | **0** | **0** | **0** |
| Parcial Transconjugant | **10** | **0** | 39 | 17 | 31 | 30 | 24 | 35 | **0** | 25 | **0** | 31 |
| 211*^b^* | **0** | **0** | 20 | **0** | **0** | **14** | 22 | 27 | **0** | **0** | **0** | **0** |
| Partial transconjugant | 24 | 24 | 41 | 20 | 24 | 29 | 26 | 34 | **0** | **0** | **0** | **0** |
| 191 | **0** | **0** | 20 | **0** | **0** | **14** | 23 | 27 | **0** | **0** | **0** | **0** |
| Complete transconjugant | **0** | **0** | 19 | **0** | **0** | **12** | 24 | 26 | **0** | **0** | **0** | **0** |
| 58*^c^* | 20 | **13** | 33 | 17 | **0** | **0** | 23 | 28 | 16 | **0** | **10** | **0** |
| 132 | 19 | **0** | 29 | 15 | **11** | 31 | 20 | 28 | 13 | **0** | **0** | **0** |
| Complete transconjugant | 21 | **0** | 37 | 19 | **10** | 34 | 22 | 32 | 13 | **0** | **0** | **0** |
| 19 | 16 | **10** | 30 | 16 | 22 | 32 | 19 | 29 | **10** | **0** | **0** | **0** |
| Complete transconjugant | 23 | **10** | 35 | 18 | 24 | 34 | 22 | 29 | **10** | **0** | **0** | **0** |
| 44 | 18 | **0** | 30 | 16 | 24 | 32 | 19 | 27 | **10** | **0** | **0** | **0** |
| Complete transconjugant | 15 | **0** | 41 | 18 | 30 | 38 | 28 | 37 | **0** | **0** | **0** | **0** |
| 90 *^a^* | 20 | **0** | 31 | 15 | 25 | 27 | 22 | 30 | **0** | **0** | **0** | **0** |
| Complete transconjugant | 19 | **0** | 40 | 19 | 31 | 32 | 25 | 34 | **0** | **0** | **0** | **0** |
| Parcial Transconjugant | 16 | **0** | 39 | 19 | 31 | 33 | 28 | 35 | **0** | **0** | **0** | 25 |
| 113 *^a^* | 17 | **0** | 33 | 17 | 23 | 31 | 21 | 29 | **0** | **0** | **0** | **0** |
| Complete transconjugant | 17 | **0** | 36 | 17 | 30 | 35 | 23 | 28 | **0** | **0** | **0** | **0** |
| Parcial Transconjugant | 20 | **0** | 39 | 19 | 24 | 34 | 25 | 31 | **0** | **0** | **0** | 21 |
| 169 | 19 | **0** | 33 | 17 | 24 | 34 | 20 | 28 | **0** | **0** | **0** | **0** |
| Complete transconjugant | 21 | **0** | 43 | 17 | 29 | 34 | 28 | 33 | **0** | **0** | **0** | **0** |
| 200 | 19 | **0** | 32 | 18 | 27 | 27 | 23 | 30 | **0** | **0** | **0** | **0** |
| Complete transconjugant | 16 | **0** | 43 | 18 | 33 | 46 | 26 | 36 | **0** | **0** | **0** | **0** |

**SUPPLEMENTAL MATERIALS Table S1. *(Continued.)***

| **Isolate ID** | **AMC** | **AM** | **CTX** | **CF** | **C** | **CIP** | **CN** | **IPM** | **S** | **G** | **TE** | **SXT** |
| --- | --- | --- | --- | --- | --- | --- | --- | --- | --- | --- | --- | --- |
| 202 | 24 | **0** | 35 | 19 | 25 | 38 | 22 | 32 | **0** | **0** | **0** | **0** |
| Complete transconjugant | 20 | **0** | 43 | 19 | 30 | 33 | 26 | 34 | **0** | **0** | **0** | **0** |
| 203 *^a^* | 20 | **0** | 31 | 16 | 26 | 35 | 22 | 32 | **0** | **0** | **0** | **0** |
| Complete transconjugant | 16 | **0** | 42 | 19 | 32 | 40 | 27 | 35 | **0** | **0** | **0** | **0** |
| Parcial Transconjugant | 20 | **0** | 43 | 19 | 33 | 34 | 25 | 34 | **0** | **0** | **0** | 23 |
| 212 | 22 | **0** | 31 | 17 | 22 | 26 | 21 | 31 | **0** | **0** | **0** | **0** |
| Complete transconjugant | 18 | **0** | 44 | 19 | 34 | 50 | 27 | 36 | **0** | **0** | **0** | **0** |
| 233 *^a^* | 16 | **0** | 34 | 15 | 28 | 33 | 23 | 30 | **0** | **0** | **0** | **0** |
| Complete transconjugant | 15 | **0** | 39 | 18 | 31 | 36 | 28 | 35 | **0** | **0** | **0** | **0** |
| Parcial Transconjugant | 20 | **0** | 42 | 18 | 31 | 34 | 27 | 35 | **0** | **0** | **0** | 25 |
| 71 | 22 | 18 | 35 | 18 | 27 | 24 | 19 | 29 | 14 | **0** | **0** | **0** |
| Complete transconjugant | 24 | 20 | 32 | 18 | 25 | 25 | 21 | 28 | 14 | **0** | **0** | **0** |
| 253 | 22 | 19 | 33 | 15 | 23 | 28 | 19 | 29 | 16 | **0** | **0** | **0** |
| Complete transconjugant | 23 | 19 | 32 | 17 | 25 | 28 | 22 | 30 | 16 | **0** | **0** | **0** |
| 50 | 22 | 21 | 32 | 16 | 24 | 39 | 22 | 25 | **0** | **0** | **0** | **0** |
| Complete transconjugant | 21 | 21 | 39 | 21 | 22 | 33 | 25 | 32 | **11** | **0** | **0** | **0** |
| 226 | 21 | 18 | 36 | 18 | 29 | 30 | 22 | 31 | 0 | **0** | **0** | **0** |
| Complete transconjugant | 23 | 22 | 43 | 22 | 25 | 32 | 26 | 35 | **0** | **0** | **0** | **0** |
| 241 *^a^* | 23 | 19 | 33 | 20 | 23 | 33 | 24 | 30 | **0** | **0** | **0** | **0** |
| Complete transconjugant | 25 | 23 | 42 | 20 | 31 | 34 | 27 | 35 | **0** | **0** | **0** | **0** |
| Parcial Transconjugant | 22 | 21 | 40 | 19 | 29 | 31 | 25 | 33 | **0** | **0** | **0** | 25 |
| 102 | 18 | **0** | 33 | **14** | **0** | **0** | 23 | 31 | **11** | **0** | **0** | **0** |
| Complete transconjugant | 18 | **0** | 33 | **14** | **0** | **0** | 22 | 29 | **10** | **0** | **0** | **0** |

Antimicrobial compounds used are abbreviated as follows: AMC, amoxicillin-clavulanate; AM, ampicillin; CTX, cefotaxime; CF, cephalotin; C, chloramphenicol; CIP, ciprofloxacin; GM, gentamicin; IPM, imipenem; S, streptomycin; G, sulfisoxazole; TE, tetracycline; SXT, trimethoprim-sulfamethoxazole.

Twenty-three isolates transferred their complete multireresistant pattern to the receptor bacteria (complete transconjugant), *^a^*six isolates transferred both partial resistance (defined as resistance to some antimicrobials of the complete multiresistance pattern of the donor isolate) and total resistance, and *^b^*one isolate transferred partial resistance pattern only (partial transconjugant). *^c^*One isolate was not conjugated. Values in bold indicate resistance.
