# Supplementary figures and images for: Diverse Commensal Escherichia coli Clones and Plasmids Disseminate Antimicrobial Resistance Genes in Domestic Animals and Children in a Semirural Community in Ecuador
Source: mSphere. 2019 May 22;4(3):e00316-19. doi: 10.1128/mSphere.00316-19 (PMC6531886; doi:10.1128/mSphere.00316-19)

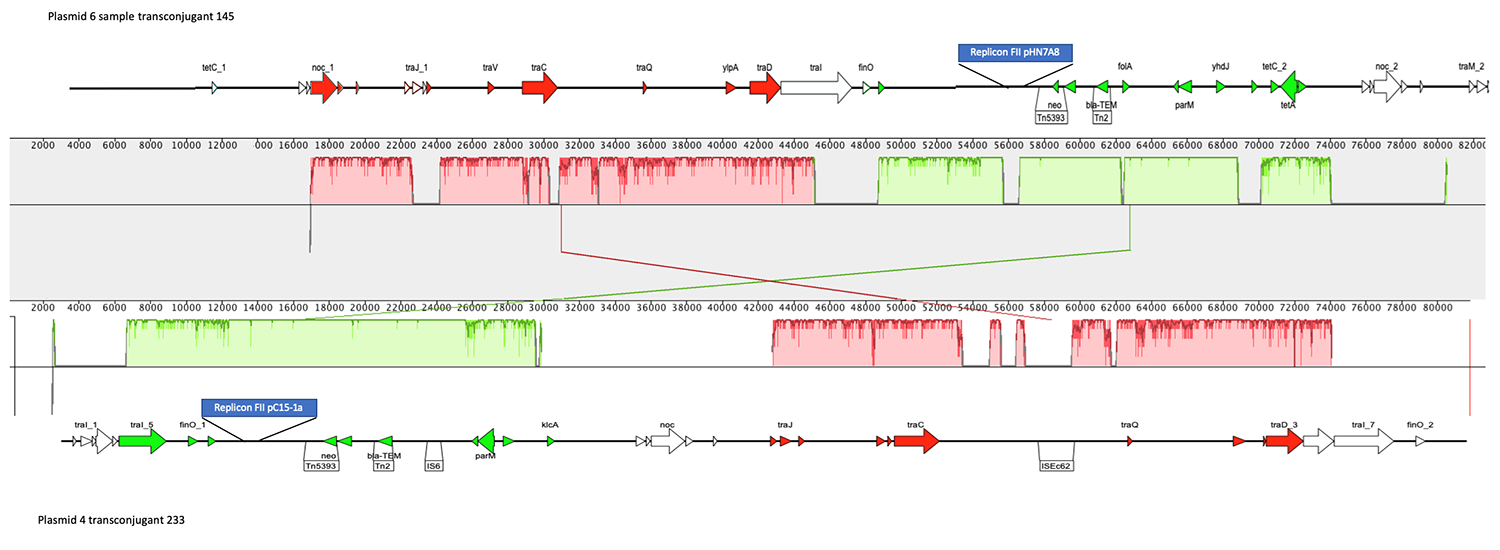

Supplement: FIG S1 [file mSphere.00316-19-sf001.tif]
